# Modelling Health in University Students: Are Young Women More Complicated Than Men?

**DOI:** 10.3390/ijerph18147310

**Published:** 2021-07-08

**Authors:** Éva Bíró, Sándor Kovács, Ilona Veres-Balajti, Róza Ádány, Karolina Kósa

**Affiliations:** 1 Department of Public Health and Epidemiology, Faculty of Medicine, University of Debrecen, 4032 Debrecen, Hungary; adany.roza@med.unideb.hu; 2 Department of Economical and Financial Mathematics, Faculty of Economics and Business, Institute of Statistics and Research Methodology, University of Debrecen, 4032 Debrecen, Hungary; kovacs.sandor@econ.unideb.hu; 3 Department of Physiotherapy, Faculty of Public Health, University of Debrecen, 4032 Debrecen, Hungary; balajti.ilona@sph.unideb.hu; 4 MTA-DE Public Health Research Group, University of Debrecen, 4032 Debrecen, Hungary; 5 Department of Behavioural Sciences, Faculty of Medicine, University of Debrecen, 4032 Debrecen, Hungary; kosa.karolina@med.unideb.hu

**Keywords:** biopsychosocial model of health, life course perspective, university students, multivariate analysis, wellbeing, physical activity, social support

## Abstract

The biopsychosocial model of health in a limited life course perspective was tested among students in higher education using data from a nationwide cross-sectional survey of students on track to become teachers in Hungary. Health determinants were grouped into categories of biological, psychological, and social determinants and arranged in a temporal manner from childhood to the present. The model was tested by canonical correlation analysis followed by multivariate analysis of covariance. One composite outcome of health and six determinant groups were examined out of a total of 24 variables in both genders. Separate sets of health determinants were identified for men and women. The health of men was determined by fewer variables that were more proximal in time, more centred around physical activity, and less influenced by social relations. As opposed to that of men, women’s health was influenced by age; determinants were grouped around the ingestion of various substances and social support. In contrast to men, the health of women seemed to be more obviously multifactorial. The study supports the usefulness of the biopsychosocial model of health in research. The best fit models provided evidence for the importance of gender awareness when designing public health interventions aimed at students.

## 1. Introduction

Ever since Engel called attention to the inadequacies of the biomedical model of health and proposed a more comprehensive, biopsychosocial (BPS) perspective [1], the biopsychosocial model of health has found increasing acceptance. The use of its practical application has been shown in various fields of clinical medicine [2,3,4], health psychology [5,6], and public health [7,8]. According to the World Health Organization (WHO), the BPS model provides a coherent view of different perspectives of health from a biological, individual and social point of view [7]. Therefore, the BPS constitutes the basis on which the International Classification of Functioning, Disability, and Health (ICF)—the WHO framework for measuring health and disability at both individual and population levels accepted in 2001—rests. Some researchers have called for the revision of the ICF model [9,10], however, the use of the general biopsychosocial model as a dynamic multilevel explanatory framework is still relevant [11].

Nevertheless, the BPS model continues to have its share of criticism on theoretical and practical grounds alike in the scientific literature. Of all fields of health care, psychiatry and mental health care have not embraced the BPS model or have even argued against it [12,13]. The argumentation is based on the grounds that its boundaries are not sufficiently specific, especially in terms of the psychological approach to be used [14]; that it is not an appropriately developed model at all, and no proper methods are available for investigating its dimensions [15]. Further counterarguments are that its three main domains are not sufficiently integrated [5], and there is not enough empirical evidence to support it [16].

A life course approach represents an attempted synthesis of models of disease causation integrating biological and social risk processes [17]. This approach allows the study of long-term effects on disease risks of physical and social exposures during gestation, childhood, adolescence, young adulthood, and later adult life. It includes studies of the biological, behavioural, and psychosocial pathways that operate across an individual’s life course, as well as across generations, influencing the development of diseases [18]. Life course epidemiology builds and tests theoretical models that postulate pathways linking exposures across the life course to health outcomes later in life [17].

**Figure 1:**
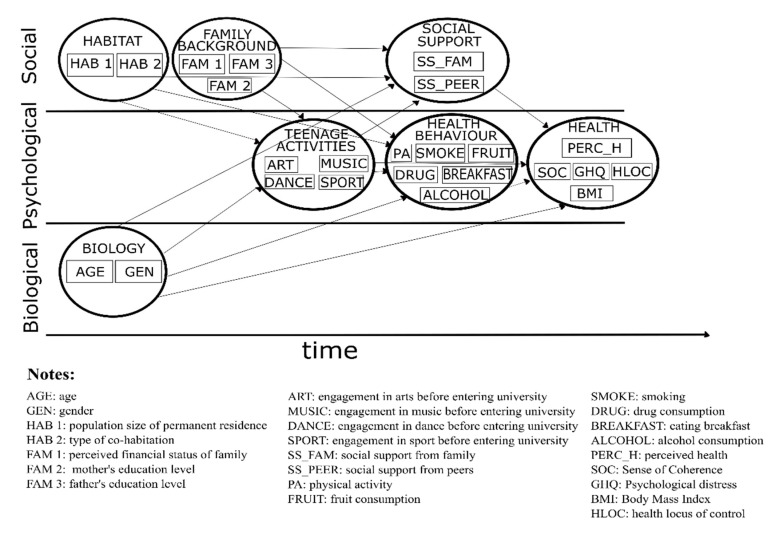
The hypothetical life course model of health for students in higher education.

The aim of the present study was to test the validity of the BPS model of health from a life course perspective [18] by using data from a nationwide survey of students in Hungary. In our hypothesised model shown in Figure 1, a life course model of health (including its mental and physical aspects together) was constructed by dividing exposures into biological, psychological (teenage activities and lifestyle), and social (sociodemographic variables and social support) domains in accordance with the BPS model. Investigated exposures were arranged in a temporal (based on their occurrence from childhood to present) and inter-related manner as recommended [17]. Mental health, as an outcome, was measured by various psychological constructs such as the dynamic feeling of confidence measured by sense of coherence, psychological distress, and health locus of control. Physical health was approximated by body mass index, and perceived health, including physical and mental aspects, was used as a summary measure of health. The model was tested separately for women and men since some of the outcome measures have been known to differ by gender.

## 2. Materials and Methods

### 2.1. Participants

Sampling was done by stratified systematic sampling as described elsewhere in detail [19]. The sampling frame included students of 27 faculties of the six largest universities and colleges in Hungary (*N* = 30,901 students) of whom 5% were systematically sampled. 1059 questionnaires were received, yielding a response rate of 68.6%. Of those, 1010 questionnaires (65.4%) were eligible for analysis. The mean age of the students was 23.3 years (age range: 20–49; 97.2% below the age of 30; standard deviation: 2.88); 67% of the respondents were women.

### 2.2. Tools of Measurement

The questionnaire included domains on demographic (age, gender, population size of permanent residence, type of cohabitation meaning whom the respondent lived with), socioeconomic indicators (parents’ education, perceived financial status of family), health, health behaviour (physical activity, diet, smoking, alcohol and drug use), and social support from family and peers. Social support was measured by seven questions, each on a 1–3 scale. Overall sum scores ranged from 7 to 21 (Cronbach’s alpha = 0.846). The maximum score of 21 indicated no lack of social support, scores of 18 to 20 indicated a moderate lack of social support, and scores of 17 showed that individuals perceived a severe lack of social support. One item addressed perceived support from peers at the university. Questions not cited separately were adapted from the tool of the Hungarian National Health Interview Survey (HNHIS) 2003 [20] for the purpose of comparisons.

A set of four questions asked respondents about their extracurricular activities before entering higher education, such as dance, art, music, and any kind of sporting activity outside of school-based mandatory physical education. These latter questions were piloted in an earlier small-scale survey (unpublished).

The following variables were used to assess health:

#### 2.2.1. Perceived Health

Perceived health was assessed by a standard question with five response categories from “very bad” to “very good” as recommended by the WHO [21]. Responses were analysed as categorical variables.

#### 2.2.2. Psychological Distress (PD)

PD was measured by the 12-item General Health Questionnaire (GHQ). Items are answered on a 1 to 4-point Likert scale. Cases were detected by scoring in the simplest manner, the so-called usual (0-0-1-1) method which assigns a score of 1 to each existing symptom, while symptoms absent are scored 0. Summarising these yields a score between 0 and 12; notable psychological distress was indicated by scores above 4 [22]. Responses were analysed as binary variables. The Cronbach’s alpha of the scale was 0.869.

#### 2.2.3. Sense of Coherence (SoC)

Antonovsky’s SoC was quantified by the abbreviated 13-item Orientation to Life questionnaire [23]. Items are answered on a 7-point Likert scale, where the scores of the items 1, 2, 3, 7 and 10 have to be reversed before calculating the total sum score, which varies between 13 and 91. Higher scores indicate stronger levels of sense of coherence. Scores were analysed as continuous variables. The Cronbach’s alpha of the scale was 0.829.

Hungarian versions of GHQ and SoC were validated and published earlier [24].

#### 2.2.4. Health Locus of Control (HLoC)

HLoC was approximated by the question of “how much can you do for your health” with four categories of answer from “nothing” to “very much”. Responses were analysed as categorical variables.

#### 2.2.5. Body Mass Index (BMI)

The respondents were asked about their body height without shoes and their body weight without clothes. These data were used to calculate BMI with the usual formula (weight (kg)/height (m^2^)), which was analysed as a continuous variable.

### 2.3. Data Collection

Since students at six universities in six cities were included, nationwide data collection was needed for the research. Since neither mailed paper questionnaires alone nor online questionnaires alone were predicted to provide a sufficiently high response rate [25], the optimal method for data collection had to be found. Details of this process were described elsewhere [19]. Briefly, a pilot study was carried out to choose the method which produced the highest response rate in the most cost-effective manner. Based on its results, a combination of postal and Internet-based questionnaires was used for data collection to which a small up-front gift was enclosed, and conditional incentives upon response were promised.

### 2.4. Construction of the Model

Data were analysed by Stata 10.0 and SPSS 22 software. Since the distribution of the values of psychological distress (test value = 0.122; *p* < 0.001) and sense of coherence (test value = 0.043; *p* = 0.001) were not normal according to the Kolmogorov–Smirnov test, medians were used for point estimation and interquartile range (IQR) for interval estimation of these variables. Scores for SoC and psychological distress were stratified by gender, and medians were compared by the Kruskal–Wallis test. The chi-square and Fisher’s exact tests were used to investigate gender differences in terms of categorical variables.

Health as a latent outcome variable was defined by five measured variables of which three related to mental health: SoC as a measure of a dynamic feeling of confidence, GHQ as an approximate measure of psychological distress, and health locus of control approximating the perception of how much control the respondent had over his/her health. Body mass index was used as a measure of physical health, and perceived health was included as a subjective measure of general health. Explanatory variables were grouped into the following latent variables: health behaviour (current physical activity, alcohol, drug consumption, fruit consumption, smoking, eating breakfast); social support (from family and from peers); teenage activities (engagement in sport, arts, dance or music before entering higher education); family background (father’s and mother’s education, economic status of family); habitat (population size of permanent residence, type of cohabitation in terms of whom the respondent lived with); and biology (age, self-identified gender). Latent variables were arranged on a time axis (time) from childhood to the present on which habitat, family background, and biological factors were defined as (from the present) most distal determinants. Teenage activities were expected to impact upon current health behaviour and social support, whereas all these together thought to impact upon current health status (HEALTH) as shown in Figure 1. We tested this model, including all data using the methods described below, separately for men and women.

At first, canonical correlation analysis was carried out to select variables to be included in the model. Outcome variables constituted the first set, and all other variables were included in the second set in order to determine relationships among these two sets.

The next step was to fit a multivariate analysis of covariance (MANCOVA), keeping in mind its limitation, namely that it is not appropriate for establishing hierarchical relations. The reason to apply MANCOVA was that it fit our purpose better as several groups could be compared with respect to more outcome variables at the same time. We also wished to remove the effect of some concomitant variables called covariates. The effect of a covariate could serve to reduce the error variance of the outcomes. On the other hand, MANCOVA provided the advantage of modelling differences in variances and means over time between groups. The steps of modelling are described in detail below.

### 2.5. Multivariate Analysis of Covariance (MANCOVA)

The full dataset with 1010 subjects was used for the analysis. Only those outcomes and groups of determinant variables, as well as covariates, were included that remained significant in the previous analysis. The data matrix was first examined for missing data and outliers on the outcomes using the Missing Value Analysis procedure in SPSS 22. Boxplots of the outcomes on the seven groups of determinant variables were also explored. Overall, 116 cases were removed as outliers so that the final sample size was reduced to 894. The proportion of missing values was less than 5% for almost all variables and was replaced by the mean. The assumptions of MANCOVA were tested by a series of univariate analyses of variance (ANOVA). Levene’s test was used to check the equality of variance, and Box’s M test was used for the homogeneity of covariance assumption. In light of the rather large number of groups, multivariate normality could be assumed because of the central limit theorem. The assumption of low measurement error of the covariates was tested by calculating Cronbach’s alpha coefficient. Interaction terms between covariates and groups of determinants were checked by performing univariate covariance analyses and were considered significant at 5%. F-ratio, Wilks’s lambda, and eta squared were used to interpret the results of MANCOVA [26].

## 3. Results

Of those who responded, 65.2% rated their health as very good or good. There was no significant difference in terms of perceived health between men and women (*p* = 0.470).

Regarding psychological distress (GHQ), almost one-quarter (23.6%) of the respondents scored above the cut-off value (4) that indicates unfavourable mental status. The proportion of women with notable psychological distress was significantly higher compared to men (26.5% vs. 17.6%; *p* = 0.002). The median score of men was significantly more favourable, reflecting lower distress than that of women (*p* < 0.001). The median for SoC was 62 points (IQR: 16, min: 21, max: 87), with no significant difference between men and women (*p* = 0.862). There was a marked gender difference in psychological distress, therefore, the model of health was analysed separately for men and women.

### 3.1. Variable Selection for Modelling “Health”

Based on the results of the canonical correlation analysis, two major dimensions were formulated. The first dimension may be designated as “mental health” that was mainly correlated with drugs (except smoking), social support, and sport in teenage years. The perceived financial status of the family and maternal education were also influential on this dimension (mental health). The second dimension approximated “physical health” that showed a strong relationship with age, drugs (except marijuana) and dancing in teenage years. Based on these results, other variables were omitted from further investigations. Wilk’s lambda statistic was 0.601, and the F-statistic also proved to be significant (*p* < 0.001) of all canonical correlations (r1 = 0.518; r2 = 0.282).

As described above, health as a group of outcome variables (HEALTH in Figure 1) was approximated by body mass index (BMI), sense of coherence (SoC), psychological distress (GHQ), health locus of control (HLOC), and perceived health (PERC_H). A reasonable but modest correlation was found among these outcome variables except for body mass index, so this was excluded from the final model. Health locus of control was also excluded from the outcomes in the final model as its correlation with covariates was weak, and it also violated the assumption of equality between-group variances (*p* < 0.001; F = 1.679). Accordingly, sense of coherence, psychological distress, and perceived health were included in the final model.

In addition, six groups of determinant variables (variables of health behaviour such as smoking and fruit consumption, social support from family and support from peers, use of sedatives with or without a prescription and physical activity), and four covariates such as age, teenage experience with dance, sport, and arts were defined. After the inclusion of covariates, multivariate outcomes became much stronger in most cases, and some of the error variances were also reduced. The assumption of homogeneity of the covariances was fulfilled (Box’s M: 339.11; F(234,6233) = 1.064; *p* = 0.243). Reliability analysis for covariates yielded a reasonably high Cronbach’s alpha coefficient (0.768).

### 3.2. Result of MANCOVA for Men

As can be seen from Figure 1, the hypothetical model posited the determinants of health at different time points (see “time” axis). Living conditions, family background, age, and gender were set as the most distal (farthest from the present) determinants, followed by teenage activities (activities before entering university). Health behaviour and social support were experienced while at university. All these impacted the latent variable of actual health as approximated by the three measured variables described above.

All the most distant determinant variables disappeared from the multivariate analysis of covariance for men (R squared = 0.767), leaving only sport and dance as teenage activities the most distant determinants, of which sport impacted indirectly (through interaction with actual physical activity), dance impacted directly on health (Table 1).

**Figure 2:**
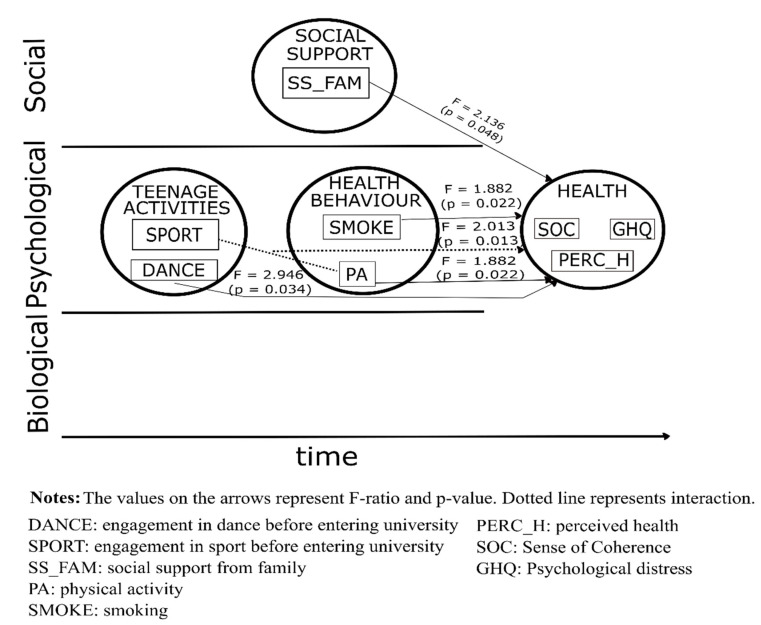
Best fit model for the relationship between health and its determinants in male students.

Actual physical activity, smoking, dance during teenage years and social support from their family had a significant positive impact on health measured by sense of coherence, psychological distress, and perceived health in male students (Figure 2).

### 3.3. Result of MANCOVA for Women

MANCOVA yielded a different result for women (R squared = 0.551). Of the most distant determinant variables, age had a significant direct impact on current health. Dance during teenage years had a significant direct impact on current health, while teenage sport impacted only indirectly through peer support and smoking. Current use of sedatives without prescription, fruit consumption, smoking, social support from family and peers alike had a direct, significant impact on health (Table 2).

**Figure 3:**
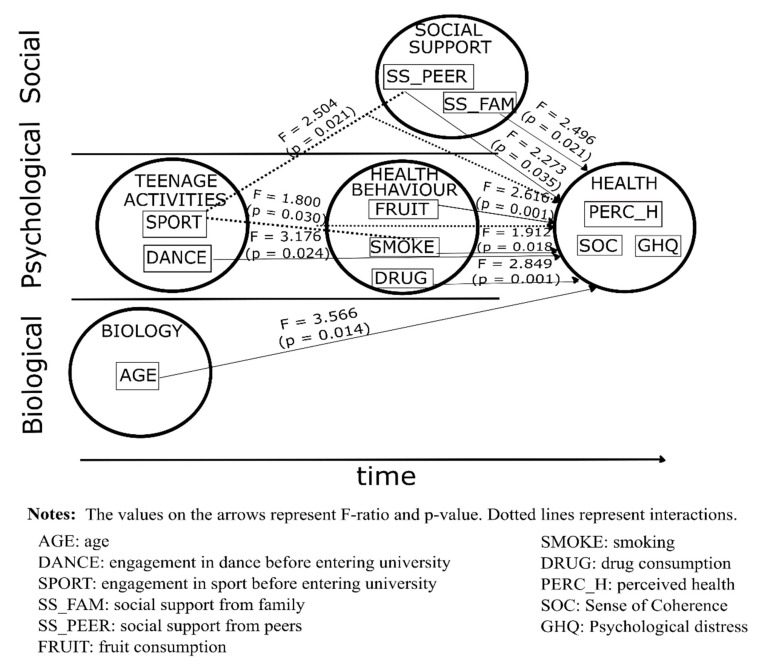
Best fit model for the relationship between health and its determinants in female students.

All determinants—except current physical activity—found significant in men were also significant in women as well, but in addition, age, social support from peers, fruit consumption, and use of sedatives without prescription (drug use) also remained in the model. In contrast to male students, female students’ health seemed to be more obviously multifactorial (compare Figure 3 to Figure 2).

## 4. Discussion

The biopsychosocial model of health with a limited life course perspective was found to be a useful point of departure to investigate the relationship between various health determinants and health outcomes in a large sample of university students by multivariate analysis of covariance. Considerably different sets of health determinants were identified for the two genders. Male students’ health was determined by fewer variables that are more proximal in time (closer to the present), more centred around physical activity, and less influenced by social relations. Female students’ health, as opposed to that of men, was influenced by age; more determinants were grouped around the ingestion of various substances and social support. Our final models did not contradict our hypothetical model (shown in Figure 1) and were in line with other findings regarding gender differences in the genesis of health [27,28]. Regarding the possible reasons why are there fewer variables involved in men’s health and more that influence women’s health, only speculation can be offered. Gender is a socioeconomic determinant of health according to the World Health Organization [29]. Women’s quality of life and opportunities have still been lagging behind that of men, which is why gender equality and women’s empowerment has been one of the 17 sustainable development goals of the United Nations [30]. Disadvantage and inequality due to being a woman results in greater difficulties to satisfy needs that tend to call for greater efforts to mobilise resources. Therefore, a gender approach can be helpful in a number of areas, for example, it can accelerate the prevention of noncommunicable diseases [31,32,33,34,35].

One of the strengths of our model derives from the fact that determinants of health were measured by a rather comprehensive set of variables. Another strength is the inclusion of positive (sense of coherence) and negative (psychological distress) measures of mental health along with perceived health, a reliable measure of general health [36]. The BPS model in a life-course perspective allowed the creation of an easy-to-comprehend visual guide (model). The generalizability of our results is supported by the rather large dataset from a nationwide sample of students on track to become teachers at the largest universities of the country, but limitations following from self-reported assessment tools have to be taken into consideration when interpreting the results.

Optimal modelling should include longitudinally generated temporal [17] and hierarchical [37] data. One shortcoming is the cross-sectional nature of data collection in our study. However, this was countered by specific items that related to earlier periods in the students’ lives. Data were not representative for all students in higher education in Hungary, but since the effect size measures of the model were acceptable, especially in light of the complexity of the latent outcome variable (health) and the high number of explanatory variables, general conclusions may be drawn. The generalisability of our results should be confirmed in other populations, including older persons. Men and women were not different in terms of sense of coherence, but—though women had greater social support and used more coping techniques—a greater proportion of women were overstressed compared to men. Having a wide range of coping techniques is of special importance for students preparing to become teachers because teaching—along with working in prisons and being a police officer—is one of the most stressful occupations in modern times [38]. The difference between the genders in terms of social support is in line with previous studies, where perceived social support from family or friends was more important for women compared to men [39]. Health behaviour, reflecting a particular combination of more or less effective coping techniques [40], has been shown to be a strong predictor of morbidity and mortality [41,42]. Interventions to change health behaviour at universities need to be targeted, for which our results offer a practical guide in addition to arguing for the usefulness of the biopsychosocial model of health in research aimed at uncovering the relationship between health determinants and outcomes. We hope that our study will be one of those that help “turn meaningful epidemiologic questions into studies that provide useful epidemiologic answers” [43], especially in terms of whether gender-specificity or gender-equality in health-promoting interventions are needed and how to develop proper interventions based on evidence [44]. Answers for these questions will be needed to achieve Goal 5 specifying gender equality of the Sustainable Development Goals of the United Nations [30].

## 5. Conclusions

The positive effects of social support on health, along with gender differences in health, are well known, but less is known about the specifics of gender differences on health behaviour among university students. A multivariate model of the determinants of health in a life course perspective was investigated with data obtained from college students at major Hungarian universities. Notable differences between health determinants of men and women were uncovered. Male students’ health is determined by a fewer number of variables that are more proximal (closer to the present), more focused on physical activity, and less influenced by social relations. The health of female students is influenced by more determinants, age being one of them; determinants are related to oral consumption of various substances and social support. Our final models are in agreement with our hypothetical model and the findings of others, adding further evidence to the notion that gender differences exist in health determinants that should be taken into account when planning interventions.

## Figures and Tables

**Table 1:** Effects of health determinants on indicators of health in men.

Variable	Sense of Coherence ^1^	Psychological Distress ^2^	Perceived Health ^3^	Overall Effect on the Multivariate Outcome	Partial Eta Squared ^4^
	F-ratios from the Tests of between Subject Effects			*p*-Value	According to Wilks’ Lambda
Intercept	71.254 **	62.273 **	65.218 **	**<0.001**	0.558
Social support from peers at the university	0.702	0.234	0.328	0.855	0.005
Social support from family, friends	3.528 *	0.757	0.258	**0.048**	0.031
Use of sedatives with prescription	0.199	0.367	0.919	0.937	0.005
Use of sedatives without prescription	0.160	0.770	0.725	0.957	0.007
Fruit consumption	2.058	1.666	1.150	0.378	0.021
Physical activity	1.506	1.143	2.262 *	**0.022**	0.052
Smoking	0.693	1.167	2.490 *	**0.022**	0.052
Arts	0.000	1.843	2.258	0.306	0.015
Dance	1.615	2.079	8.679 **	**0.034**	0.035
Sport	0.317	0.442	0.034	0.724	0.005
Age	0.058	0.021	0.699	0.814	0.004
Social support from peers × sport	1.550	1.780	0.252	0.302	0.015
Smoking × sport	0.314	1.178	1.075	0.507	0.019
Physical activity × sport	1.492	2.625 *	1.551	**0.013**	0.039
Social support from family, friends × age	2.024	0.845	0.193	0.341	0.014

**Notes**: Significant overall effects are marked in bold. ^1^: R squared for sense of coherence = 0.331; ^2^: R squared for psychological distress = 0.227; ^3^: R squared for perceived health = 0.297; ^4^: overall R squared = 0.767; * significant at *p* < 0.05; ** significant at *p* < 0.01.

**Table 2:** Effects of health determinants on indicators of health in women.

Variable	Sense of Coherence ^1^	Psychological Distress ^2^	Perceived Health ^3^	Overall Effect on the Multivariate Outcome	Partial Eta Squared ^4^
	F-Ratios from the Tests of between Subject Effects			*p*-Value	According to Wilks’ Lambda
Intercept	82.360	130.549	42.212	**<0.001**	0.401
Social support from peers at the university	4.827 **	0.183	0.764	**0.035**	0.012
Social support from family, friends	1.626	6.084 **	2.011	**0.021**	0.013
Use of sedatives with prescription	1.294	0.999	2.791 *	0.149	0.012
Use of sedatives without prescription	4.761 **	2.545 *	3.085 *	**0.001**	0.020
Fruit consumption	2.094	3.658 **	4.500 **	**0.001**	0.023
Physical activity	2.698 *	1.887	1.831	0.084	0.014
Smoking	1.819	1.320	2.830 *	**0.018**	0.017
Arts	1.295	1.432	0.101	0.565	0.004
Dance	0.074	7.438 **	0.078	**0.024**	0.017
Sport	0.078	0.882	2.119	0.364	0.006
Age	7.353 **	1.377	5.580 *	**0.014**	0.019
Social support from peers × sport	0.529	4.288 *	1.754	**0.021**	0.013
Smoking × sport	1.783	1.007	3.579 **	**0.030**	0.016
Physical activity × sport	2.211	1.289	0.405	0.378	0.010
Social support from family, friends × age	0.263	4.182 *	1.241	0.135	0.009

**Notes:** Significant overall effects are marked in bold. ^1^: R squared for sense of coherence = 0.331; ^2^: R squared for psychological distress = 0.227; ^3^: R squared for perceived health = 0.297; ^4^: overall R squared = 0.767; * significant at *p* < 0.05; ** significant at *p* < 0.01.

## Data Availability

The data supporting the findings of this study are available from the corresponding author upon reasonable request.
